# The lipopolysaccharide-transporter complex LptB_2_FG also displays adenylate kinase activity *in vitro* dependent on the binding partners LptC/LptA

**DOI:** 10.1016/j.jbc.2021.101313

**Published:** 2021-10-19

**Authors:** Tiago Baeta, Karine Giandoreggio-Barranco, Isabel Ayala, Elisabete C.C.M. Moura, Paola Sperandeo, Alessandra Polissi, Jean-Pierre Simorre, Cedric Laguri

**Affiliations:** 1 Université Grenoble Alpes, CNRS, CEA, IBS, Grenoble, France; 2 Dipartimento di Scienze Farmacologiche e Biomolecolari, Università degli Studi di Milano, Milan, Italy

**Keywords:** cell wall, lipopolysaccharide (LPS), ABC transporter, adenylate kinase, NMR, AK, adenylate kinase, IM, inner membrane, LPS, lipopolysaccharide, Lpt, lipopolysaccharide transporter system, LptA_m_, monomeric version of LptA, OM, outer membrane, SMA, styrene-maleic acid, STD, saturation transfer difference, TBS, Tris-buffered saline, TM, transmembrane

## Abstract

Lipopolysaccharide (LPS) is an essential glycolipid that covers the surface of gram-negative bacteria. The transport of LPS involves a dedicated seven-protein transporter system called the lipopolysaccharide transport system (Lpt) machinery that physically spans the entire cell envelope. The LptB_2_FG complex is an ABC transporter that hydrolyzes ATP to extract LPS from the inner membrane for transport to the outer membrane. Here, we extracted LptB_2_FG directly from the inner membrane with its original lipid environment using styrene-maleic acid polymers. We found that styrene-maleic acid polymers–LptB_2_FG in nanodiscs display not only ATPase activity but also a previously uncharacterized adenylate kinase (AK) activity, as it catalyzed phosphotransfer between two ADP molecules to generate ATP and AMP. The ATPase and AK activities of LptB_2_FG were both stimulated by the interaction on the periplasmic side with the periplasmic LPS transport proteins LptC and LptA and inhibited by the presence of the LptC transmembrane helix. We determined that the isolated ATPase module (LptB) had weak AK activity in the absence of transmembrane proteins LptF and LptG, and one mutation in LptB that weakens its affinity for ADP led to AK activity similar to that of fully assembled complex. Thus, we conclude that LptB_2_FG is capable of producing ATP from ADP, depending on the assembly of the Lpt bridge, and that this AK activity might be important to ensure efficient LPS transport in the fully assembled Lpt system.

Gram-negative bacteria possess a double membrane system delimiting an aqueous space, the periplasm. Although the cytoplasmic inner membrane (IM) is a canonical phospholipid bilayer, the outer membrane (OM) is asymmetric with its outer layer mostly composed of the lipopolysaccharide (LPS) glycolipid (1). These molecules are surface exposed, contribute to the high impermeability of the OM, and play a role in immune response, pathogenesis, and drug resistance (2). Maintenance of the OM asymmetry is essential for bacterial viability, and a continuous flow of LPS molecules needs to cross the periplasm and reach the external layer to keep up with the cell growth. A system of seven essential proteins, the lipopolysaccharide transport system (Lpt), is dedicated to trafficking LPS across the cell envelope. These proteins (LptA-G) are found in all cellular compartments (IM, periplasm, and OM), and in the periplasm, they associate through jellyroll domains (present in LptF, LptC, LptA, and LptD) to form a continuous periplasmic bridge connecting the IM and OM (3, 4).

LptB_2_FG IM complex is an ABC transporter in which LptB_2_ represents the nucleotide-binding domain component that binds and hydrolyzes ATP to generate mechanical force to transport LPS. Upon ATP binding, LptB dimerizes in a closed structure, and ATP hydrolysis relaxes the dimer into an open conformation. The conformational changes are transmitted to LptFG through coupling helices and allow LPS extraction (5). LptB_2_FG associates with LptC *via* two distinct interactions: (i) LptF/LptG interact with LptC transmembrane (TM) domain, which contributes to the formation of the cavity that accommodates LPS into the complex and regulates LptB_2_FG ATPase activity (5) and (ii) LptF and LptC jellyroll domains associate in the periplasm. LptA jellyroll then bridges LptB_2_FGC to the LptD/E OM complex that ultimately assembles the LPS to the OM outer leaflet (3).

The superfamily of ABC transporter proteins comprises more than 500 members and supports traffic of metabolites and molecules through ATP hydrolysis (6). Several prokaryotic ABC transporters (MsbA, TmrAB, and LmrA) couple another reaction in their ATPase domain, adenylate kinase (AK) (7). AK catalyzes a phosphotransfer reaction between two ADP molecules producing ATP and AMP with no energy consumption (8). MsbA in particular translocates LPS across the IM before its transport by LptB_2_FG (9, 10). The additional active site for the AK has been suggested to be located proximal to the ATP binding site because the reaction requires both ADP molecules close by in space (11).

LptB_2_FG was extracted directly from the *Escherichia coli* IM with styrene-maleic acid (SMA) polymers without the use of detergents. ^1^H-NMR on nucleotides showed that the SMA–LptB_2_FG complex has not only ATPase activity but also AK activity, and both activities are regulated by the assembly of LptB_2_FG with LptC and LptA partners. Point mutations were introduced in the LptB_2_FG complex and in the isolated LptB to address their effect on ATPase and AK activity by NMR and locate the AK site.

## Results

### LptB_2_FG extracted from the IM by SMA polymers displays ATPase and AK activity

**Figure 1:**
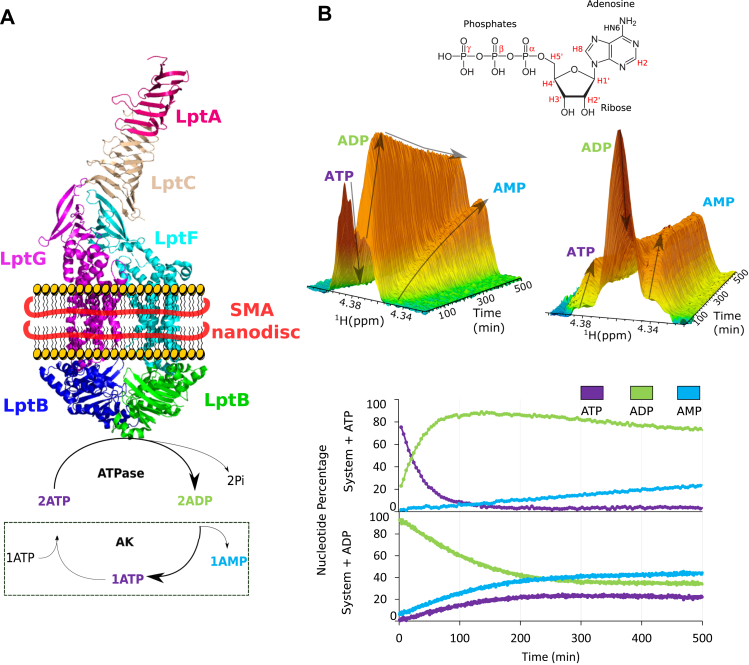
**LptB_2_FG in SMA nanodiscs shows ATPase and AK activity.***A*, representation of the LptB_2_FGCA (with ΔTM-LptC and LptAm) system in SMA nanodiscs with its updated enzymatic cycle. *B*, real-time kinetics of LptB_2_FGCA observed by ^1^H-NMR with either ATP (*left* 16 scans/trace) or ADP (*right* four scans/trace) as the substrate over time with evolution of the characteristic H4′ peaks of ATP, ADP, and AMP. Nucleotide quantification along the experiment is represented (*bottom*). Lpt, lipopolysaccharide transporter system; SMA, styrene-maleic acid.

LptB_2_FG complexes from several organisms have been expressed and purified in detergent micelles (12, 13, 14, 15), reconstituted nanodiscs (5), or liposomes (16). These protocols all involve solubilization and purification of the LptB_2_FG complex in detergent micelles. The use of detergent-free protocols in the preparation of membrane protein, to maintain their original lipid environments, becomes increasingly important to ensure functionality and stability. In that aspect, the use of SMA polymers and its derivatives, which allow the direct extraction of membrane proteins from purified membranes, provides a versatile tool to study membrane proteins as close as possible to their natural environment (17, 18). The LptB_2_FG complex extraction directly from the IM of *E. coli* was tested using two SMA polymers with 2:1 and 2.3:1 SMA ratio. SMA–LptB_2_FG was successfully purified in nanodiscs of about 10-nm diameter only with SMA 2:1 polymer and with the expected 2:1:1 LptB–LptF–LptG stoichiometry (Fig. S1). The LptB_2_FG ATP hydrolysis was checked in presence of LptC and LptA, its periplasmic partners, using NMR to probe the reaction (Fig. 1*A*). The monomeric version of LptA (LptA_m_) and the soluble version of LptC (ΔTM-LptC), which are able to sustain activity and cell viability (4, 19, 20), were added to SMA–LptB_2_FG, and ATP hydrolysis was followed by real-time ^1^H-NMR (Fig. 1*B*). NMR is a spectroscopic technique that provides resonance frequency of active nuclei, in our case protons, depending on their chemical environments (21). It can discriminate between ATP and ADP as several ^1^H shift frequencies upon phosphate loss, here, hydrogen H4′ on the ribose (Fig. 1*B*). SMA–LptB_2_FG shows ATP hydrolysis, with fast disappearance of peak characteristics of ATP and concomitant appearance of ADP-specific peaks in the NMR spectrum. The rate of ATP hydrolysis is 6.5 mol of ATP consumed/min/mol of LptB. After the initial rapid accumulation of ADP due to ATPase activity, ADP level decreases at the same rate as the appearance of a new set of NMR peaks (8.60 ppm in the H8 region and 4.39 ppm in H4′ region). This suggests the conversion of ADP into another species over time and its ^1^H-NMR chemical shifts suggest that it could correspond to AMP (22). ^31^P NMR spectrum was collected at the end of the reaction and showed the characteristic peak of AMP at 3 ppm (Fig. S2). The conversion of ADP into AMP is compatible with an AK reaction 2ADP ⇔ ATP + AMP, which has already been observed for several ABC transporters (7, 11). ATP was replaced by ADP as a substrate, and the reaction was followed in identical conditions in real time (Fig. 1*B*). ADP decrease is seen immediately, together with the appearance of AMP and ATP. The generation of ATP is consistent with AK activity and excludes the hydrolysis of ADP into AMP. The ATP levels observed are lower than those of AMP, as AK and ATPase activities occur simultaneously, and newly generated ATP is partly hydrolyzed back to ADP. The initial rates of AK activity are much slower than ATPase with 0.2 and 0.7 mol of AMP produced/min/mol of LptB with ATP or ADP as starting substrates (Fig. 1*B*).

### ATPase and AK activities are regulated by the assembly of the Lpt system

**Figure 2:**
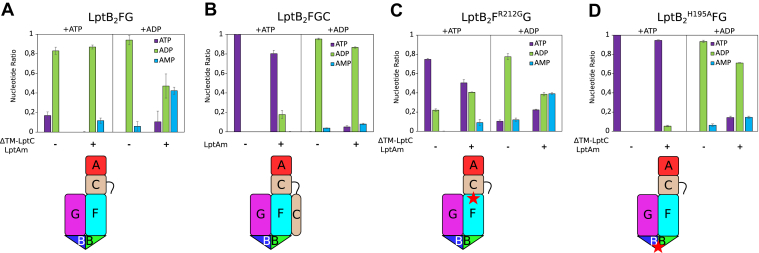
**Assembly with LptC and LptA influence ATPase and AK activity of LptB_2_FG.***Top panel*, assembly on the periplasmic or membrane level with LptC and LptA influences ATPase and AK activities of LptB_2_FG. SMA–LptB_2_FG is incubated with ATP or ADP, and nucleotide levels (ATP, ADP, and AMP) quantified by ^1^H-NMR from two or three independent experiments and the SD are shown. *A*, SMA–LptB_2_FG without/with ΔTM-LptC/LptA_m_. *B*, SMA–LptB_2_FGC complex without/with LptA_m_. *C* and *D*, SMA–LptB_2_F^R212G^G and SMA–LptB_2_^H195A^FG without/with ΔTM-LptC/LptA_m_. A schematic representation of the complexes used is shown at the *bottom panel*, with *stars* marking the location of mutations. Lpt, lipopolysaccharide transporter system; SMA, styrene-maleic acid.

LptB_2_FG ATPase activity is repressed by the TM domain of LptC (5, 16); we thus examined the ATPase and AK activities of SMA–LptB_2_FG with/without ΔTM-LptC/LptA_m_ and in complex with the full-length LptC with/without LptA_m_ (Fig. 2, *A* and *B*). For the following experiments, nucleotides were quantified by NMR at the end of incubation (Fig. S3). SMA–LptB_2_FG displays less ATPase and AK activity in the absence of ΔTM-LptC and LptA_m_. Proper assembly of the periplasmic part of LptC and its complex with LptA (20) is thus important in stimulating both ATPase and AK activity. SMA–LptB_2_FGC containing full-length LptC has no ATPase activity, but the repression is relieved in the presence of LptA_m_ (Fig. 2*B*). Similarly, SMA–LptB_2_FGC has little AK activity (Fig. 2*B*), which is increased by the addition of LptA_m_. AK activity and ATPase activities are thus both stimulated by the assembly of the LptB_2_FGCA complex without the TM LptC segment. In intact LptB_2_FGC, ATPase and AK are activated by assembly with LptA.

### Variants in periplasmic LptF domain and in cytoplasmic LptB differently affect AK activity

A number of mutations in LptB_2_FG were studied for their effect on the complex activity, either biochemically or for their *in vivo* functionality (23). Two SMA–LptB_2_FG complexes carrying already characterized mutations were purified, one on the LptF periplasmic side and one in the ATPase domain (LptB). The LptF^R212G^ variant can overcome the absence of the essential LptC protein and allows cell viability (24). R212 is located in the LptF periplasmic domain at the interface with LptC and is in the path of the LPS flow (15). SMA–LptB_2_F^R212G^G has impaired ATPase activity when compared with WT complexes (Fig. 2*C*), and ΔTM-LptC and LptA_m_ stimulate ATPase and AK activities (Fig. S3). With ADP as the substrate, SMA–LptB_2_F^R212G^G with and without ΔTM-LptC and LptA_m_ show similar production of AMP as WT complexes (Fig. 2*C*). R212G mutation, although impairing the ATPase, does not affect the AK, suggesting different regulation mechanisms of the two activities.

**Figure 3:**
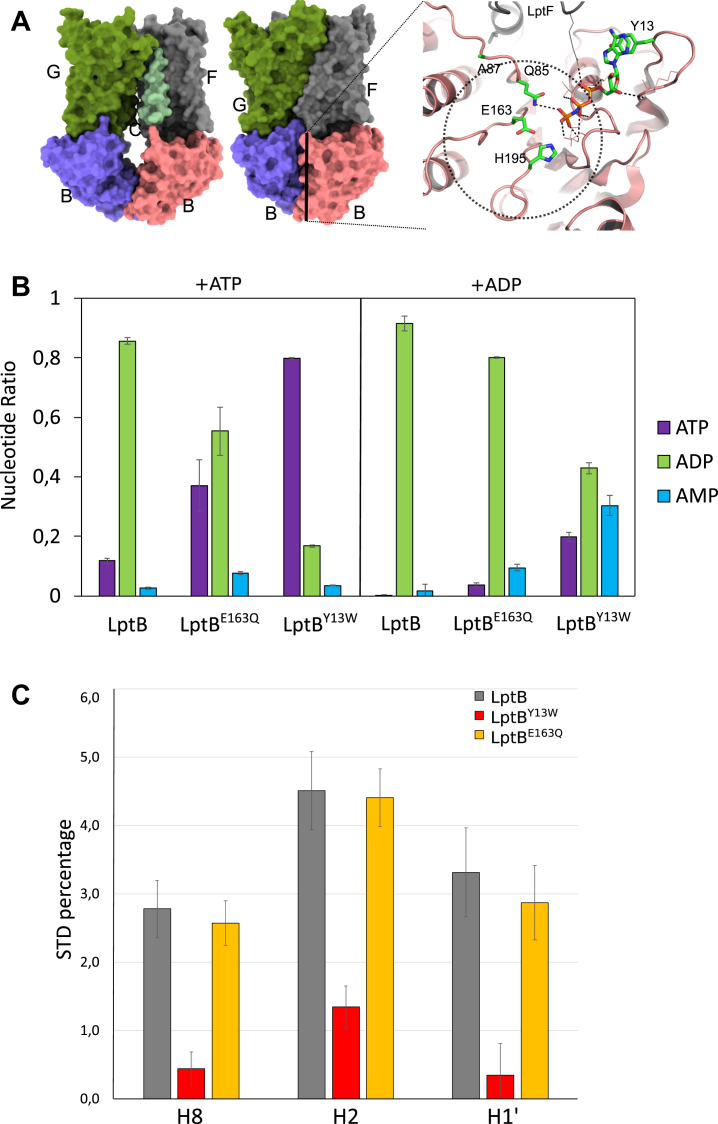
**Mutations in LptB stimulate adenylate kinase activity.***A*, structures of LptB_2_FGC in open (apo-form; PDB ID: 6S8N) and closed conformation (with bound ATP analog; PDB ID: 6S8G). The jellyroll domains were not resolved in these structures and the LptC transmembrane helix in the open conformation. The *right panel* shows the ATPase site with putative location of the second ADP in the AK reaction (*dashed line area*). The residues mutated in this study are shown. *B*, ATPase/AK of LptB_2_ and LptB_2_^E163Q^ and LptB_2_^Y13W^ mutants, with ATP or ADP as the substrate. The nucleotide levels (ATP, ADP, and AMP, in color code) were detected by the ^1^H-NMR experiment in two independent experiments, and the SD is shown. *C*, saturation transfer difference (STD) experiments of LptB_2_ and LptB_2_^E163Q^ and LptB_2_^Y13W^ on ADP-βS. The three resonances shown are from H8, H2, and H1′ on the adenosine of ADP-βS. The error bars represent two times the SD of the NMR experiments’ noise. AK, adenylate kinase; Lpt, lipopolysaccharide transport system.

LptB H195 is involved in γ phosphate binding of ATP (Fig. 3*A*), and the LptB^H195A^ variant has decreased ATPase activity in the isolated LptB and is deleterious for cell growth (23). SMA–LptB_2_^H195A^FG has almost knocked out ATPase activity, even in the presence of ΔTM-LptC and LptA_m_. AK activity is still activated by ΔTM-LptC/A_m_ but significantly decreased compared with the WT complex (Figs. 2*D* and S3). Alterations in the ATPase binding site of LptB affect the ATPase activity and AK activity, but the latter to a lesser degree.

### Mutations in the A and B loops of LptB stimulate AK

The location of the AK site is shared with the ATPase site (25, 26). One ADP molecule occupies the ATP-binding site, whereas the other must be presented “in line” with the first one for the phosphotransfer to occur. Current models for AK suggest the second ADP substrate molecule to be located around the Q-loop (11) (Fig. 3*A*). The location of the second ADP molecule was probed in LptB using mutagenesis on the LptB-isolated protein. In addition to the H195A mutation in the H motif (or switch region), the other mutations were inserted in canonical ABC motifs, specifically at the A loop (Y13W), B loop (E163Q and 163A), and Q loop (A87Q) (Fig. 3*A*).

Purified soluble LptB WT and variant proteins were incubated with either ATP or ADP, and the nucleotides were quantified by ^1^H-NMR at the endpoint of the experiment (Fig. S4). LptB, similarly to the SMA–LptB_2_FG complex (without ΔTM-LptC and LptA_m_), displays ATPase activity and little AK activity (Fig. S5). All mutations introduced in LptB decrease ATPase activity, and E163A is catalytically fully inactive, with no ATPase or AK activity. Two LptB variants show increased AK activity, E163Q and more significantly Y13W, the latter accumulating AMP at levels similar to SMA–LptB_2_FG/ΔTM-LptC/LptA_m_ (Fig. 3*B*). To assess how the binding of nucleotides relates to the activity of LptB, saturation transfer difference (STD) experiments were recorded on WT and LptB variants. STD is an NMR technique that allows observation of the free ligand in transient binding to a protein (27). The protein is selectively saturated, and part of the saturation is transferred to the ligand during binding, and a decrease of intensity is observed on the free ligand. STD on ADP by WT LptB shows saturation on protons H2, H8, and H1′ of ADP, showing significant interaction with LptB. The LptB^E163Q^ variant shows no difference of STD with respect to the WT. STD measurement of the Y13W variant with ADP is not possible because AK activity is too fast relative to the experimental time (Fig. S6). ADP-βS, which is not a substrate of LptB, showed STD similar as ADP with the WT and LptB^E163Q^ variant (Fig. S6). LptB^Y13W^, on the other hand, showed a highly reduced STD (Figs. 3*C*, S6 and S7). A reduction in STD can be ascribed to a reduced affinity of LptB^Y13W^ for ADP-βS, with a reduced residence time of the ligand, or on the contrary to a reduction in *k*_off_ leading to less saturation of the free ligand. A Y-W mutation in this position has already been found to reduce ATPase activity and ATP binding in other ABC transporters (28, 29). We thus conclude that the LptB^Y13W^ variant increase in AK activity can be assigned to a reduction in the affinity for ADP, likely in the canonical ATPase-binding site considering the position of Y13 (Fig. 3*A*). Apart from the E163A mutant, largely impaired in ATPase and AK activities, we could find no mutation that selectively affects AK activity, suggesting that a defined binding site for the second ADP molecule does not exist in LptB.

## Discussion

Understanding bacterial mechanisms that coordinate envelope assembly is critical to harness ways to challenge persistent and clinically relevant infections. Here, LptB_2_FG and LptB_2_FGC complexes were extracted directly from the *E. coli* IM in their natural lipid context, without the need for detergent extraction. The LptB_2_FG complex ATPase activity is stimulated by interaction on the periplasmic side of LptB_2_FG with LptC and LptA and fully repressed when full-length LptC is coextracted with LptB_2_FG. The SMA–LptB_2_FG LptB subunit is also an AK. This activity is marginal until LptB_2_FG interacts with the jellyroll domains of LptC and LptA, thus forming an almost complete Lpt bridge (missing the LptD/E OM complex). Establishment of the bridge must be transmitted by conformational changes through the lipid bilayer to the cytoplasmic LptB and activate both ATPase and AK activities. The LptB_2_FGC complex that contains full-length LptC with its N-terminal TM domain has no ATPase activity and little AK activity until it interacts with LptA that increases both activities. The level of activity of LptB_2_FGC is much lower because of the presence of the TM LptC domain. This domain interacts with the LPS into the LptB_2_FG complex and is more dynamic in LptB_2_FG bound to ATP analogs (5). This could suggest a role of TM-LptC in the synchronization of LPS entry and ATP hydrolysis.

The Lpt system possesses several mechanisms that regulate its activity to ensure efficient LPS transport when the Lpt system is assembled. A mutant discovered in the LptF periplasmic jellyroll domain that can complement Δ*lptC* cells is particularly interesting. SMA–LptB_2_F^R212G^G ATPase activity is highly reduced compared with the WT complex, whereas AK activity is identical. This suggests that the regulation of the ATPase and AK activities by the assembly of the Lpt system is somewhat different. Nevertheless, the regulation mechanisms are currently not characterized at the molecular level.

AK reaction involves two ADP molecules, one located in the ATPase canonical site and one located nearby. Our mutagenesis study could not pinpoint the location of a binding site for the second ADP. LptB^E163Q^ showed increased AK activity, and H195A in the full complex showed reduced AK activity. Although those residues could be important for the AK activity, they are all involved in the ATPase reaction, by binding ATP γ-phosphate, the magnesium ion, or coordinating water molecules (16). Our data suggest that, on the contrary to pure AK (30), there is no well-defined binding site for the AK activity in LptB, apart from the canonical ATP binding site. The high AK activity of the LptB^Y13W^ variant cannot be easily explained. The consequence of this mutation is to strongly reduce STD, likely because of the decreased affinity/residence time of the ligand in the canonical ATPase site. This could in turn accelerate the turnover of the reaction, but we cannot exclude that this variant in isolated LptB somehow mimics the conformation of LptB_2_FG in presence of the jellyroll domains of LptC and LptA.

The exact role of the AK activity in the LptB_2_FG–ABC transporter remains unknown. In one ABC transporter, the cystic fibrosis receptor (CFTR), the AK activity was shown to be important for the transport (31). Our characterization of the AK activity was performed at a high substrate concentration (5 mM), about ten times higher than the concentration of ADP in bacteria. The AK reaction was thus tested in physiologic concentrations with a mix of ATP, ADP, and AMP (Fig. S8). In these conditions, AK activity is similar as with 5 mM ADP as the substrate (Fig. 2). Similarly, LptB_2_FG has AK activity starting with only 500 μM of ADP, the concentration found in cells (Fig. S8). Although this reaction is possible in the concentrations of nucleotides found *in vivo*, its contribution to the LPS transport has not been tested yet. Nevertheless, the capacity of the AK reaction to generate ATP, which can be used to transport LPS, at a negligible energy cost must be considered in future studies, and specifically in conditions of bacterial stress.

## Experimental procedures

### Used plasmids

The plasmids used for soluble LptB, SMA–LptB_2_FG, ΔTM-LptC, and LptA_m_ purifications were previously described: pET22-42-LptB-His8 (23) and pCDF-Duet1-LptB_2_FG (32), expressing LptB-His6/LptF/LptG (32), pQESH LptC_Δ[1–23]_ (ΔTM-LptC) (19), and pET21b-LptA_Δ160_ (LptA_m_) (20). LptB point mutations were performed by GENEWIZ using pET22-42-LptB-His8 as the template. *lptF*^*R212G*^ mutation in pCDF-Duet1-LptB_2_FG was introduced by site-directed mutagenesis using the Q5 Site-Directed Mutagenesis Kit (New England Biolabs) and AP613 (5′-ACCAGGGAACGGGCTTCGAAGGC-3′)–AP614 (5′-TGAGAGTGACGACCTGGGAG-3′) oligonucleotides. The plasmids pBAD/HisA-LptC-His (encoding LptC with a C-terminal His8 tag) and pCDF-Duet1-utLptB_2_FG (encoding untagged LptB, LptF, and LptG) were used for the purification of SMA–LptB_2_FGC. pBAD/HisA-LptC-His is a derivative of pBAD/HisA-LptC (32) obtained by replacing the *lptC* gene (cloned into *Nco*I-*Hind*III sites) with the PCR-amplified *lptC-His8* gene using pET23/42-LptC-His (4) as the template and AP656 (5′-catgccatggGTAAAGCCAGACGTTGGGTT-3′)–AP657 (5′- cccaagcttTTAGTGGTGGTGGTGGTG-3′) oligonucleotides. pCDF-Duet1-utLptB_2_FG is a derivative of pCDF-Duet1-LptB_2_FG obtained by replacing *lptB-His6* gene (cloned into *Nco*I-*EcoR*I sites) with the PCR-amplified *lptB* gene using *E. coli* MG1655 genomic DNA as the template and AP599 (5′-catgccatggCAACATTAACTGCAAAG-3′) and AP655 (5′-gagaggaattcTCAGAGTCTGAAGTCTTCCC-3′) oligonucleotides.

### Strains used and protein expression

The LptB expression was carried out in *E. coli* BL21 (DE3) strain (Novagen) and LptB_2_FG in *E. coli* C43 (DE3) strain (Novagen). The coexpression of LptB_2_FG and LptC was carried out in *E. coli* KRX cell, as described (32). LptA_m_ and ΔTM-LptC were expressed and purified in *E. coli* BL21 (DE3), as described (20). For LptB and LptB_2_FG, bacterial cells were grown in LB, supplemented with the correct antibiotic (ampicillin 100 μg ml^−1^ and spectinomycin 50 μg ml^−1^) at 37 °C, until optical density of 600 nm (OD_600_) around 0.7. For both sets of proteins, induction was performed with IPTG: for LptB_2_ proteins, with 0.1 mM at 20 °C for 16 h, whereas for LptB_2_FG proteins, with 0.5 mM at 37 °C for 3 h. The cells were harvested by centrifugation at 6000*g* for 20 min at 4 °C and frozen at −20 °C until purification. The coexpression of LptB_2_FG with LptC and cell harvesting was carried out as before, except induction that was carried out with 0.02% (w/v) L-rhamnose and 0.02% (w/v) L-arabinose.

### Purification of LptB_2_ proteins

The cells were mixed with buffer A containing 20 mM Tris HCl, 150 mM NaCl, 20% (v/v) glycerol, pH 8.0, 0.5 mM Tris(2-carboxyethyl)phosphine, and cOmplete EDTA-free protease inhibitor cocktail (Sigma) and lysed by sonication. Soluble fraction was separated by centrifugation at 10,000*g* for 20 min at 4 °C and loaded after addition of 10 mM imidazole into Ni-NTA Agarose (QIAGEN). Resin was washed with buffer A with 20 mM imidazole and eluted with buffer A with 300 mM imidazole. LptB was then purified on a HiLoad 16/600 Superdex 200 pg column (GE Healthcare) in Tris-buffered saline (TBS) (50 mM Tris HCl and 150 mM NaCl, pH 8.0) supplemented with 0.5 mM Tris(2-carboxyethyl)phosphine. LptB was concentrated with a 10-kDa cut-off Amicon Ultra Centrifugal Filter (Merck), and the sample concentration was determined by running a 15% SDS-PAGE with known concentration samples of bovine serum albumin. The yields of purified proteins for the various LptB proteins were 30, 24, 15, 5, 3, and 1 mg/l of the culture for WT, H195A, E163A, E163Q, A87Q, and Y13W, respectively.

### Purification of LptB_2_FG and LptB_2_FGC

The cells in the lysis buffer (50 mM Tris HCl, 300 mM NaCl, 1 mM MgCl_2_, pH 8.0, and cOmplete EDTA-free [Sigma]) were lysed on a microfluidizer at 15,000 psi. The cell debris are removed by centrifugation at 10,000*g* for 20 min at 4 °C, and the membranes are collected by centrifugation at 100,000*g* for 1 h at 4 °C. The membranes are resuspended in 50 mM Tris HCl, 250 mM NaCl, pH 8.0, with 0.5% SMA 25010 (Xiran SL25010 Polyscope) for 17 h at room temperature (RT). Soluble SMALP particles were obtained by ultracentrifugation at 100,000*g* for 30 min at 4 °C and loaded into a HisTrap 1-ml column equilibrated in 20 mM Tris HCl, 150 mM NaCl, and 30 mM imidazole, pH 8.0. Elution was carried out in a gradient with the previous buffer supplemented with 170 mM imidazole. Fractions containing the proteins were dialyzed against TBS (20 mM Tris and 150 mM NaCl, pH 8.0) at RT and concentrated with a 100-kDa cut-off Amicon Ultra Centrifugal Filter (Merck). The sample concentration was determined by running a 15% SDS-PAGE with known concentrations of bovine serum albumin.

### Electron microscopy

SMA–LptB_2_FG at 59 μg/ml was prepared by the negative-stain mica-carbon flotation technique. The samples were absorbed to the clean side of a carbon film on mica, stained with 2% Na_4_O_40_SiW_12_ in distilled water, and then transferred to a 400-mesh copper grid. The images were taken under low-dose conditions (<10 e^−^/Å^2^) with defocus values between 1.2 and 2.5 μm on a Tecnai 12 LaB6 electron microscope at 120-kV accelerating voltage using CCD Camera Gatan Orius 1000.

### NMR spectroscopy

The experiments were recorded on Bruker 600-, 700-, 850-, and 950-MHz spectrometers equipped with triple ^1^H, ^13^C, ^15^N resonance cryoprobes, at 25 °C for ^1^H and ^31^P and at 37 °C for real-time kinetic analysis, in TBS with 10% D_2_O. All 1D ^1^H experiments were based on the Bruker zgesgp experiment with water suppression using sculpting with gradients and 15-s interscan delay. The data were processed using TopSpin 3.5 and Ccpnmr Analysis 2.4.2. ATPase and AK activities were checked by supplying ATP or ADP as the substrate. For LptB_2_FG/C, 5 μM of complex was incubated with 5 mM of nucleotide and 1 mM MgCl_2_ in TBS. When necessary, LptC_Δ[1–23]_/and LptA_Δ160_ were added at 10 μM. These activity assays were incubated at 37 °C for 7 h, flash-frozen, and transferred to 3-mm NMR tubes to be analyzed. For LptB, 2 μM of protein was incubated with 5 mM of nucleotide/2.5 mM MgCl_2_ in TBS at 25 °C for 17 h, flash-frozen, and transferred to 3-mm NMR tubes. The STD experiments were recorded on a 700-MHz cryoprobe at 25 °C, with Bruker stddiffesgp.3 pulse sequence alternating on and off resonance at 0.5 ppm and −40 ppm, with a 60-ms spinlock to suppress protein signals. 500 μM of the nucleotide (ADP or ADPβS) is mixed with 12.5 μM LptB (WT or mutant) with 50 μM MgCl_2_ in TBS with 1.7% glycerol and 10% D_2_O.

## Data availability

All data are contained within this article.

## Supporting information

This article contains supporting information.

## Conflict of interest

The authors declare that they have no conflicts of interest with the contents of this article.
