# Supplementary material for: The lipopolysaccharide-transporter complex LptB2FG also displays adenylate kinase activity in vitro dependent on the binding partners LptC/LptA
Source: J Biol Chem. 2021 Oct 19;297(6):101313. doi: 10.1016/j.jbc.2021.101313 (PMC8633020; doi:10.1016/j.jbc.2021.101313)
Supplement: Supplemental Figures S1–S8 [file mmc1.pdf]

Supporting information for :

The lipopolysaccharide transporter complex LptB2FG also displays adenylate kinase activity in vitro dependent on binding partners LptC/LptA

Tiago Baeta, Karine Giandoreggio-Barranco, Isabel Ayala, Elisabete C. C. M. Moura, Paola Sperandeo, Alessandra Polissi, Jean-Pierre Simorre and Cedric Laguri

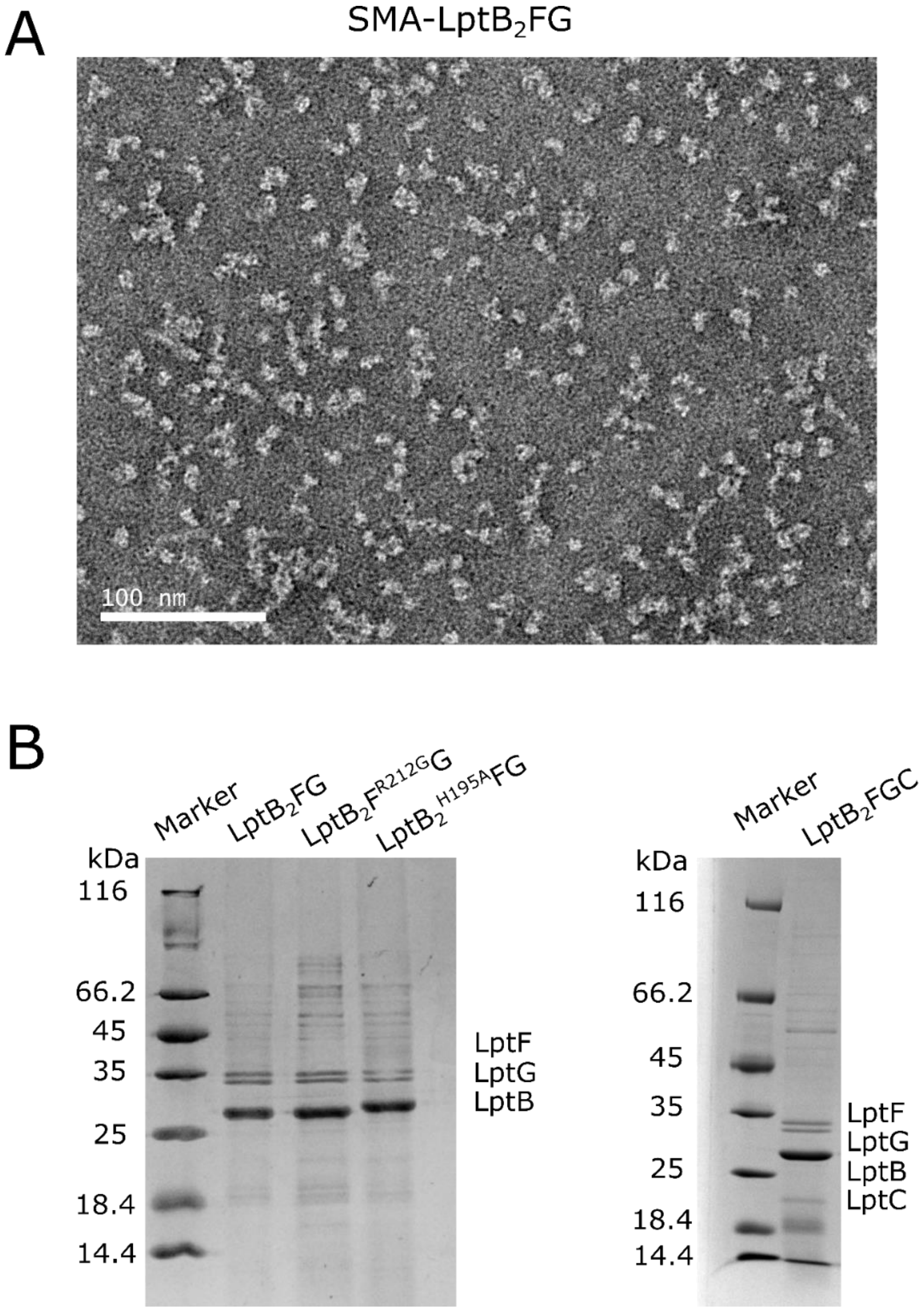

Figure S1 – (A) Representative image of Electron Microscopy negative staining of SMA-$LptB_2FG$ at 40µg/ml with Sodium Silico Tungstate staining; (B) SDS-PAGE 15% of $LptB_2FG$ and $LptB_2FGC$ complexes extracted into SMA nanodiscs. 2µg of complexes are injected.

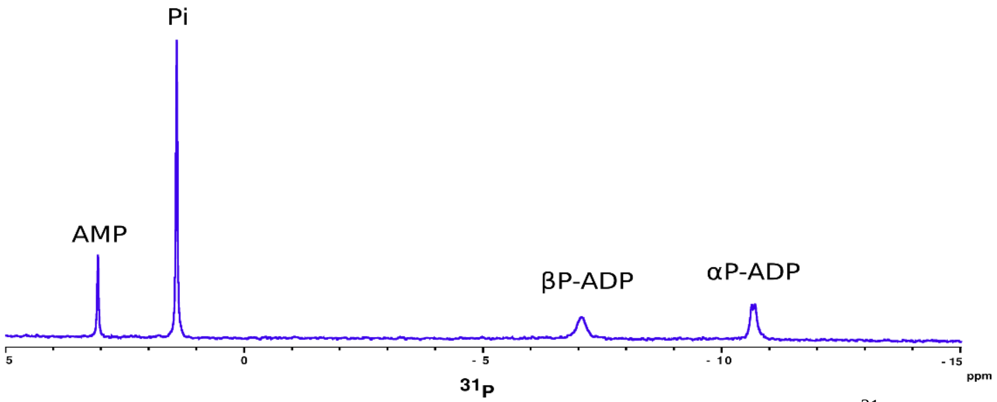

Figure S2 – $LptB_2FG$ catalyses an adenylate kinase reaction. 1D ^{31}P-NMR spectrum of $LptB_2FG/\Delta TM\text{-}C/A_m$ after incubation with 5 mM ATP, with phosphorus resonances of ADP and AMP α-phosphate displayed.

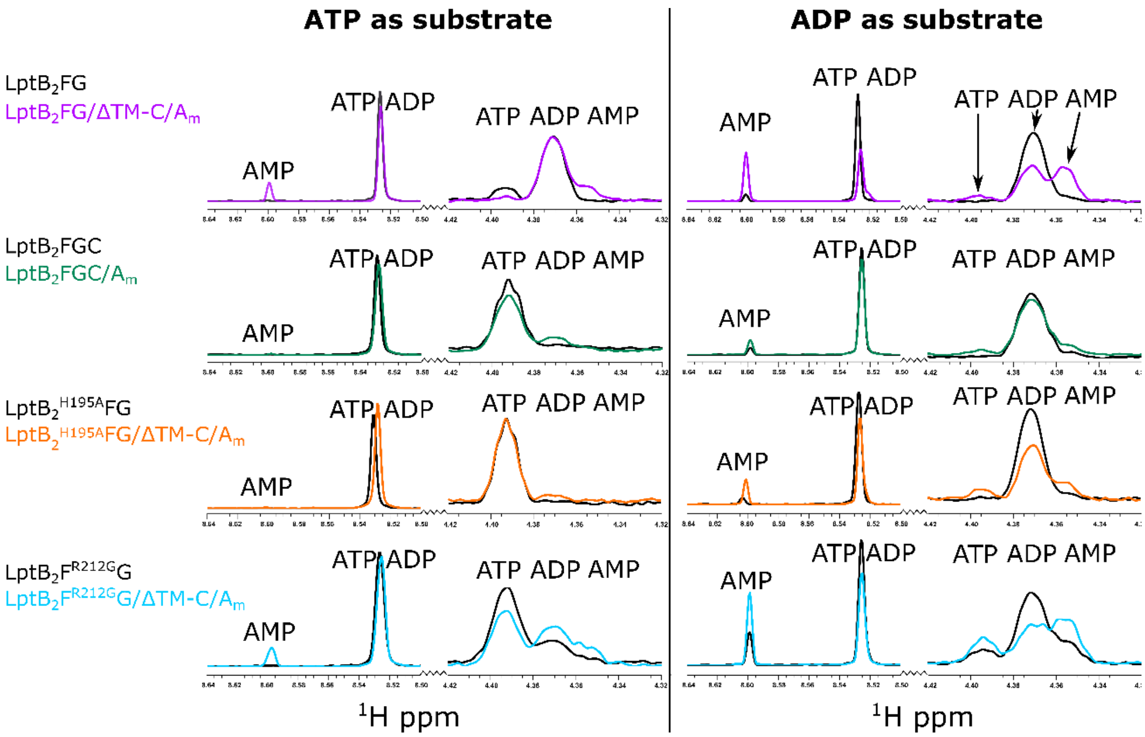

Figure S3 – 1D ^{1}H-NMR spectra showing ATPase/ AK activity of $LptB_2FG$, $LptB_2FG$/ΔTM-C/A_m, $LptB_2FGC/A_m$, $LptB_2^{H195A}FG$/ΔTM-C/Am and $LptB_2F^{R212G}G$/ΔTM-C/Am, either with ATP or ADP as substrate. Image displays zoom in the frequencies of the NMR probes used in the Adenosine H8 and Ribose H4' regions (spectrum level x4). Complex alone is displayed in black, while addition of remaining Lpt partners are displayed in colour-code accordingly, for both ATP-supplied and ADP-supplied experiments. H8 resonances from ADP and ATP are overlapped in these experimental conditions.

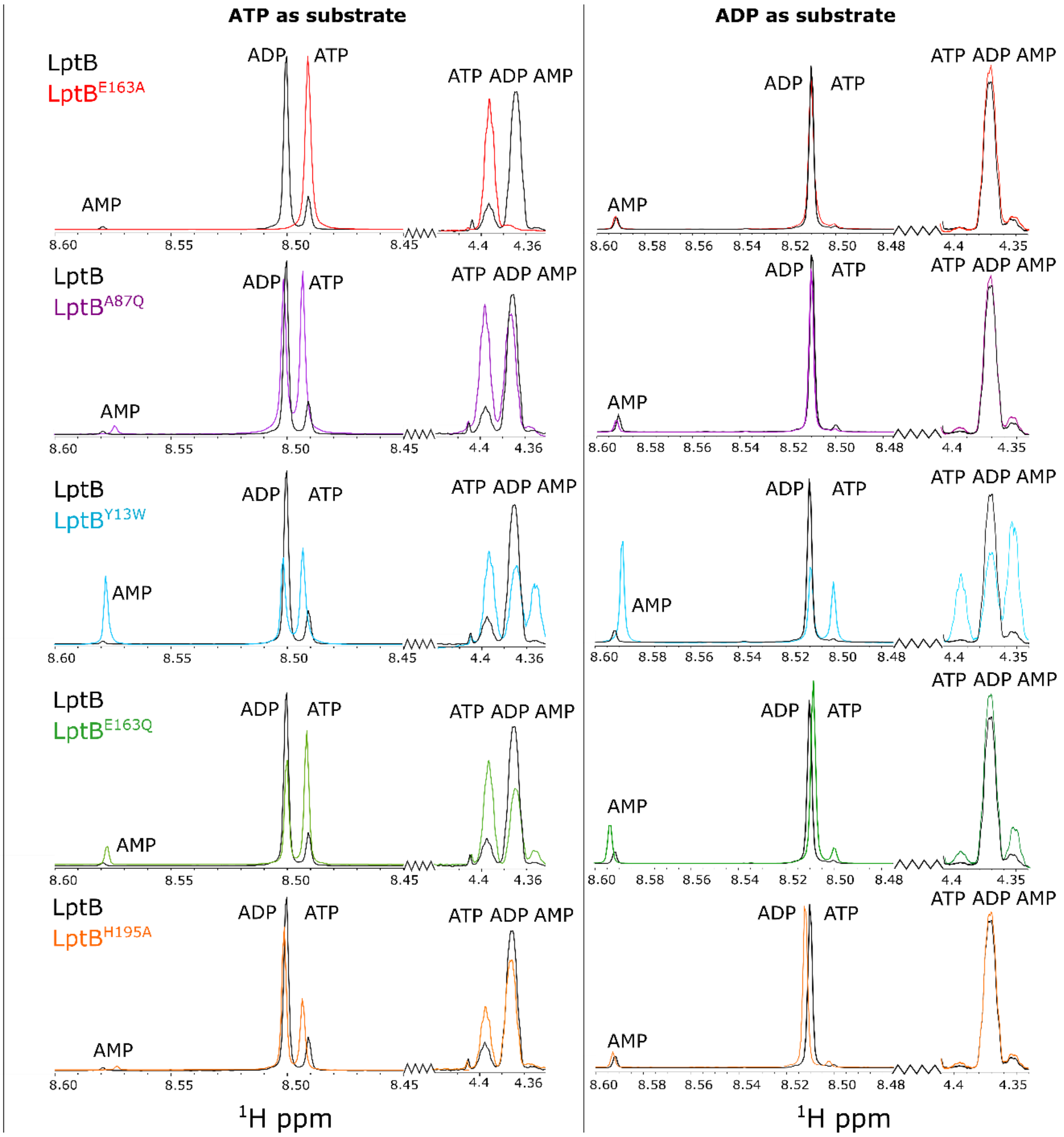

Figure S4 – 1D ^{1}H-NMR spectra showing ATPase/ AK activity of isolated wild-type LptB and variant proteins, either with ATP or ADP as substrate. Image displays zoom in the frequencies of the NMR probes used in the Adenosine H8 and Ribose H4’ resonances (spectrum level x4). Wild-type LptB protein spectrum is displayed in black, while each of the mutants are displayed in colour-code accordingly, for both ATP-supplied and ADP-supplied experiments.

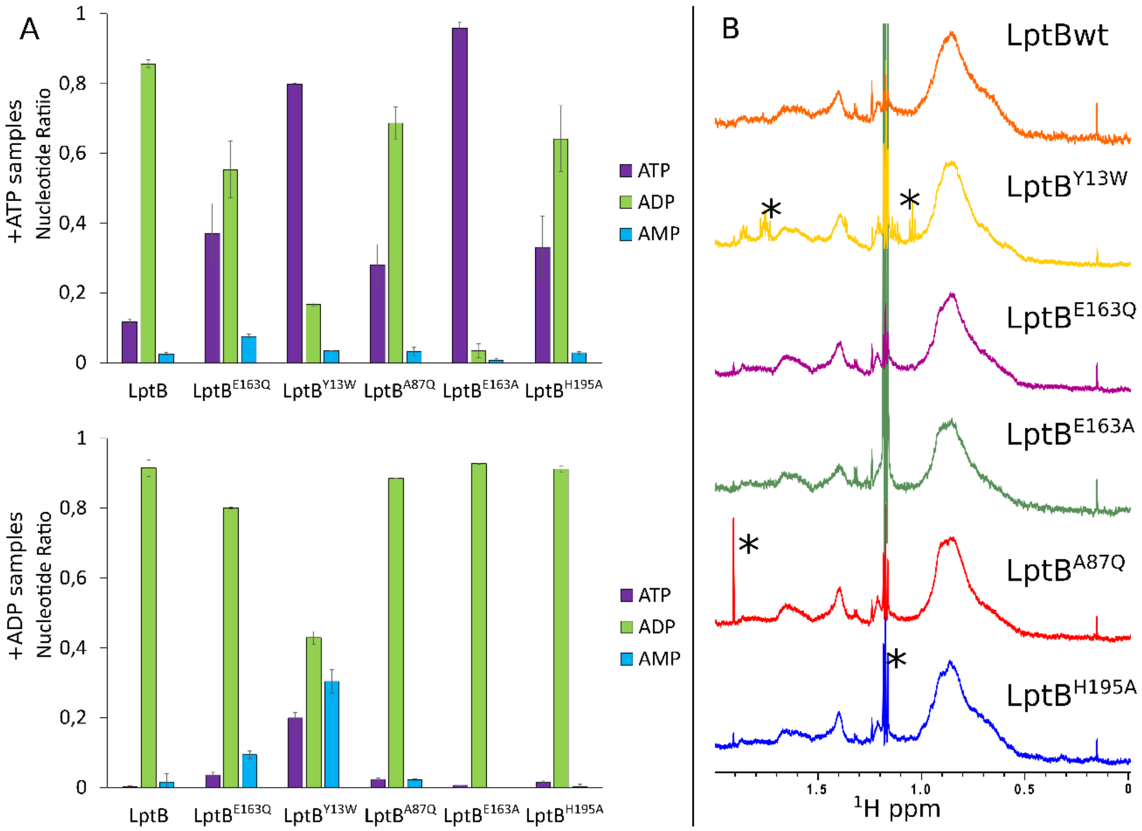

Figure S5 – ATPase and AK activities of $LptB_2$ wt, and variants in presence of either ATP or ADP, at 20°C. A) Nucleotide levels (ATP, ADP and AMP, in colour-code) were detected using a 1D ^{1}H-NMR experiment in 3 mm tubes at 20°C, extracting peak intensities for each specie in 2 independent experiments and the standard deviation is shown. B) 1D ^{1}H NMR spectra of LptB wt and mutant proteins at 30µM in TBS buffer, at 20°C, focused on the methyl region. Asterisks mark contaminants.

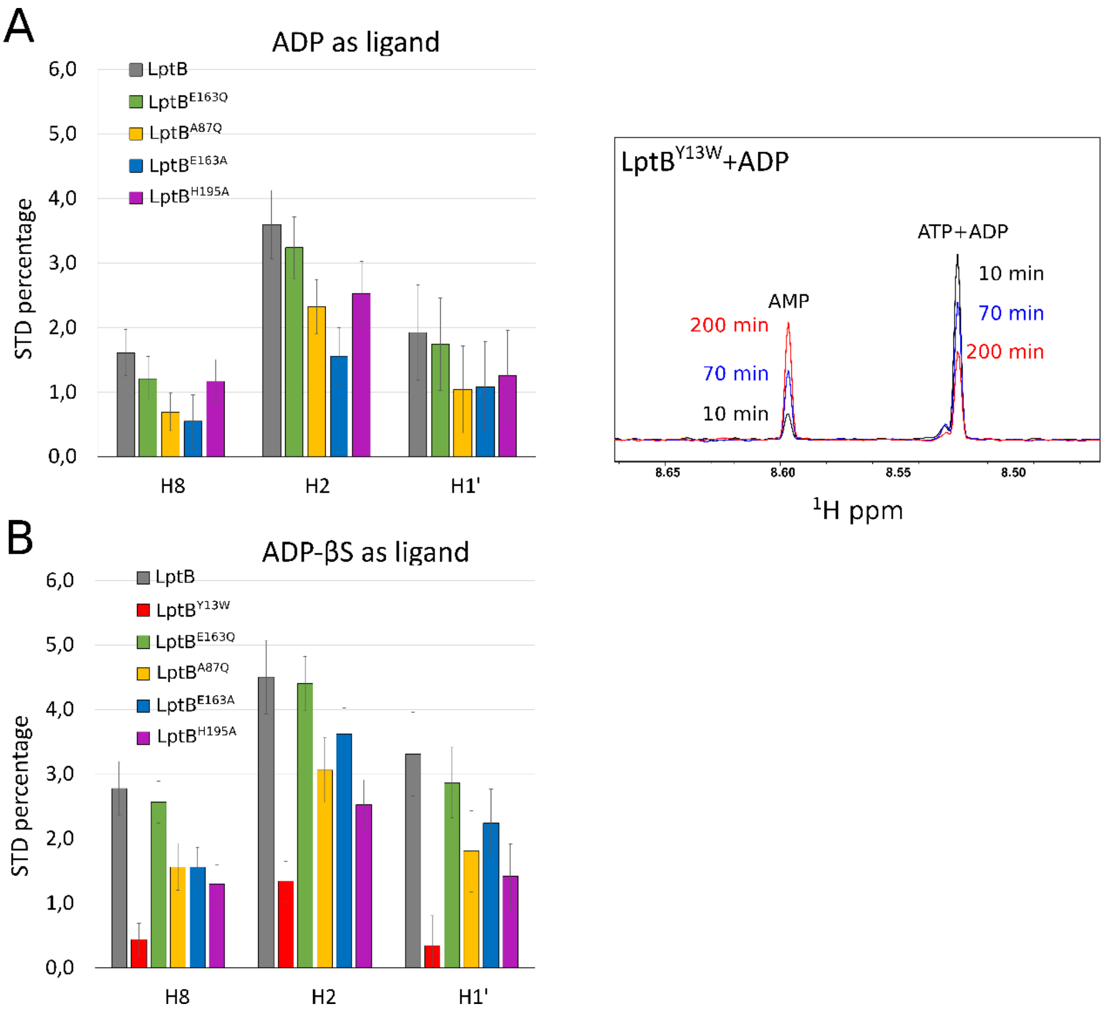

Figure S6 – Saturation Transfer Difference (STD) experiments using two ligands ADP and ADP-βS and wild-type and mutants LptB proteins: (A) STD on ADP resonances(left), except for $LptB^{Y13W}$ mutant for which AK reaction is too fast. The ^{1}H 1D NMR spectrum of $LptB^{Y13W}$ +ADP showing AK reaction with the time after tube preparation in the conditions of the STD experiment. (B) STD on ADP-βS, for LptB proteins (wt and variants in colour-code). The three resonances shown are from H8, H2 and H1' on the adenosine of ADP and ADP-βS. STD is recorded with 500µM nucleotide and 12.5µM protein concentration respectively. Data are represented as (Iref-Isat/Iref). Error bars represent two times the standard deviation of the NMR experiments' noise.

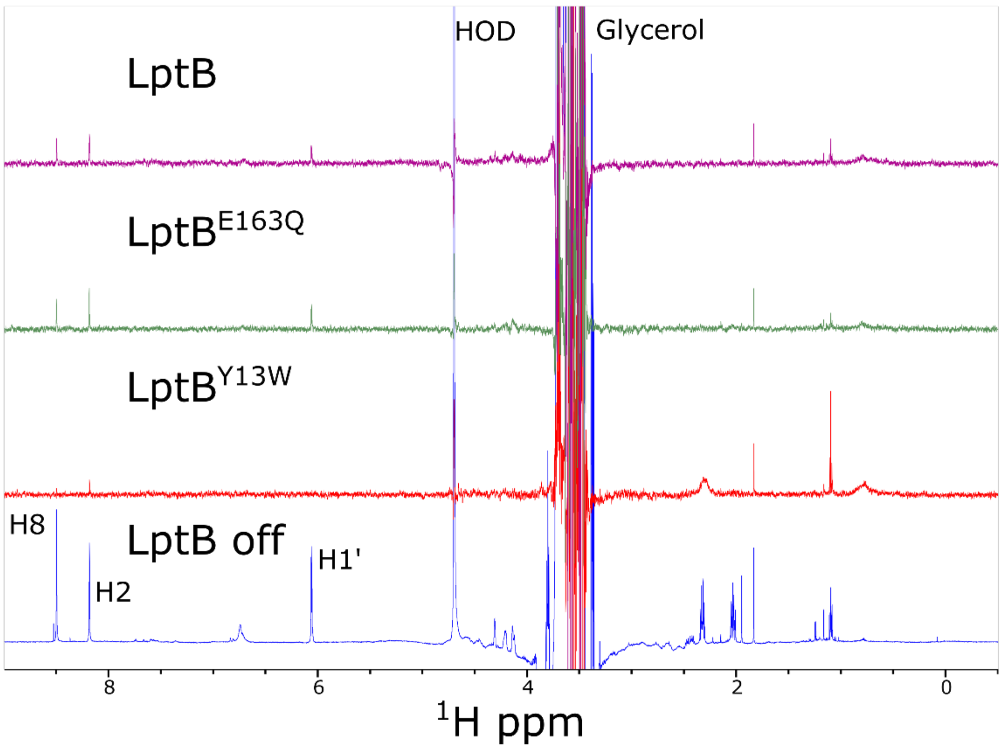

Figure S7 – Saturation transfer difference spectra of LptB, LptB^{E163Q} and LptB^{Y13W} with ADPβS. (TOP) Difference spectra of ADPβS (OFF -ON resonance) in presence of LptB, LptB^{E163Q} and LptB^{Y13W}. (Bottom) OFF resonance spectra of ADPβS in presence of LptB.

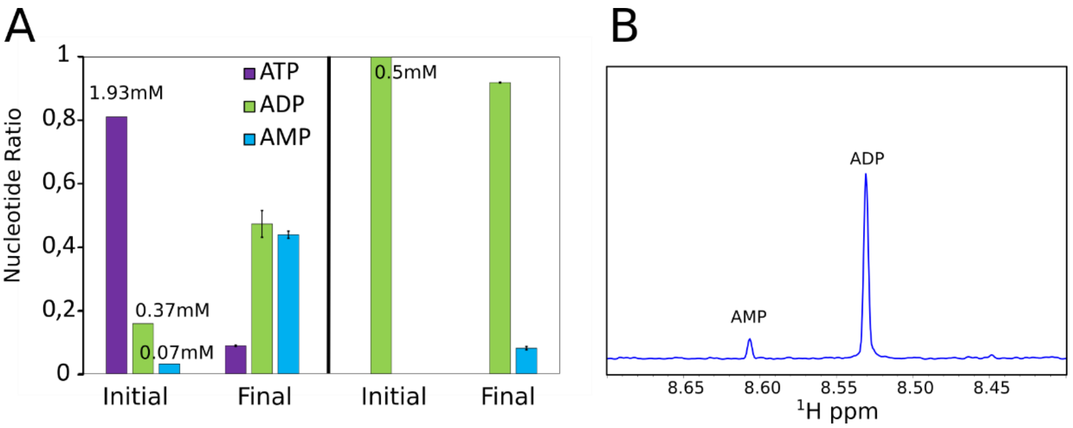

Figure S8 – $LptB_2FG$ has adenylate kinase activity at nucleotide concentrations equal to *E. coli* cytoplasmic concentrations. (A) $LptB_2FG/\Delta TM\text{-}C/A_m$ is incubated with different initial nucleotides concentrations, a mix of ATP/ADP/AMP similar as found in *E. coli* (left) and 0.5mM ADP (right) with the corresponding 1D ^1H NMR spectrum (B). Nucleotides levels (ATP, ADP and AMP) are quantified by ^1H NMR from two independent experiments and the standard deviation is shown.
